# EX-527 Prevents the Progression of High-Fat Diet-Induced Hepatic Steatosis and Fibrosis by Upregulating SIRT4 in Zucker Rats

**DOI:** 10.3390/cells9051101

**Published:** 2020-04-29

**Authors:** Amit Kundu, Prasanta Dey, Jae Hyeon Park, In Su Kim, Seung Jun Kwack, Hyung Sik Kim

**Affiliations:** 1 School of Pharmacy, Sungkyunkwan University, Suwon 16419, Korea; amitjupcl@gmail.com (A.K.); deyprasantadey@yahoo.com (P.D.); sky3640@naver.com (J.H.P.); insukim@skku.edu (I.S.K.); 2 Department of Biochemistry and Health Science, Changwon National University, Gyeongnam 51140, Korea; sjnkwack@changwon.ac.kr

**Keywords:** EX-527, fatty liver, liver fibrosis, inflammation, SIRT4

## Abstract

Sirtuin (SIRT) is known to prevent nonalcoholic fatty liver disease (NAFLD); however, the role of SIRT4 in the progression of hepatic fibrosis remains unknown. We hypothesize that EX-527, a selective SIRT1 inhibitor, can inhibit the progression of high-fat diet (HFD)-induced hepatic fibrosis. We found that SIRT4 expression in the liver of NAFLD patients is significantly lower than that in normal subjects. In this study, EX-527 (5 µg/kg), administered to HFD rats twice a week for ten weeks, reduced the serum levels of triglyceride (TG), total cholesterol, alanine aminotransferase (ALT), and aspartate aminotransferase (AST) and attenuated hepatic fibrosis evidenced by Masson’s trichrome and hepatic fat by oil red-O staining. EX-527 upregulated SIRT2, SIRT3, and SIRT4 expression in the liver of HFD fed rats but downregulated transforming growth factor-β1 (TGF-β1) and α-smooth muscle actin (α-SMA) expression. It decreased proinflammatory cytokine production and hydroxyproline levels in the serum and SMAD4 expression and restored apoptotic protein (Bcl-2, Bax, and cleaved caspase-3) expression. These data propose a critical role for the SIRT4/SMAD4 axis in hepatic fibrogenesis. SIRT4 upregulation has the potential to counter HFD-induced lipid accumulation, inflammation, and fibrogenesis. We demonstrate that EX-527 is a promising candidate in inhibiting the progression of HFD-induced liver fibrosis.

## 1. Introduction

Advanced liver fibrosis can progress to cirrhosis and lead to serious complications in liver disease. Among the liver diseases, nonalcoholic fatty liver disease (NAFLD) is the most common chronic liver disorder in Western countries [1]. The incidence of NAFLD has been rapidly increasing worldwide during the last few decades [2,3].

Furthermore, fat accumulation characterized by an increase in intrahepatic triglyceride content in the liver is a major cause of NAFLD development [3]. Elevated fasting blood glucose and obesity are risk factors for NAFLD, nonalcoholic steatohepatitis (NASH), fibrosis, and cirrhosis. The correlation between obesity and the progression of type 2 diabetes and liver fibrosis is reported [2,3,4]. Obesity is one of the key parameters for the increase in development of liver fibrosis. In order to prevent liver fibrosis, caloric restriction is suggested for obese patients, but its efficacy is challenged by poor compliance and alterations in the gut microbiome [5,6]. Thus, pharmaceutical interventions are important for NAFLD and complications such as liver fibrosis and steatosis. Therefore, treatment of obesity with suitable drug therapies will be needed to inhibit the progression of hepatic fibrosis.

The activation of hepatic stellate cells (HSCs) in liver fibrosis is an important phenomenon; however, the mechanisms remain unclear. In acute, as well as chronic liver injury, HSCs becomes activated, and they transform into myofibroblast-like cells [6,7]. The cytokines, like transforming growth factor beta (TGF-β) and PDGF, cause perpetuation of HSCs and lead to increased extracellular matrix (ECM) production [8,9]. Understanding the molecular mechanism behind HSC activation is crucial for novel antifibrotic drug development. Disrupting the gene and key molecular pathways involved in this process could suppress fibrogenesis and allow for the remodeling and regression of liver fibrosis. Additionally, the high expression of collagen-1 plays a pivotal role in liver fibrotic disorder [10,11,12]. Generally, the degree of ECM production, as well as cytokine secretion by Kupffer or inflammatory cells, directly correlate with HSCs that may be triggered during hepatic fibrosis [6,7]. TGF-β1 has a potential role in activating the c-Jun N-terminal kinase/stress-activated protein kinase (JNK)/p38 signaling cascade. The regulation of apoptosis and epithelial to mesenchymal transition (EMT) are stimulated by (JNK)/p38 along with Smad through transcription and activation factors [13,14].

The SIRT family of proteins are well connected with multicellular and physiological functions along with fatty acid and glucose metabolism, hepatic gluconeogenesis, mitochondrial function, regulation of insulin and maturation of fat cells [15,16]. It controls protein function via posttranslational modification as well as deacetylation, succinylation and malonylation [17,18]. All seven types of sirtuins (SIRT1-SIRT7) are working on same catalytic site and NAD binding domain. Each and every sirtuins have their specific cellular localization and expression. SIRT 1, 6, and 7 are located in nucleus; SIRT 3, 4 and 5 are present in the mitochondrial matrix; and SIRT2 mainly in cytoplasm [19]. Research based evidence revealed that SIRT4 is the key regulator of mitochondrial metabolic pathways, which includes insulin secretion, controlling mitochondrial ATP and regulating the apoptosis and redox pathways [20]. A recently published report revealed downregulation of most sirtuins expression in NAFLD compared with controlled groups [21]. SIRT1 expression changes under different physiological and morbid conditions. In generally, SIRT1 is decreased in conditions of chronic metabolic stress, oxidative stress, or hypoxia that drive the pathophysiology of age-related diseases including diabetes, cardiovascular, NAFLD and renal diseases.

Recently, sirtuin (SIRT) activators have been identified for their preventive function against the development of hepatic fibrosis [22,23]. SIRT1 is the most comprehensively studied in the HDAC family, which is involved in the prevention both of NAFLD and alcoholic fatty liver diseases (AFLD) [21,24,25]. In addition, SIRT1 levels were markedly decreased in mouse models of hepatic fibrosis, cultured HSCs undergoing activation, and in cirrhosis patients [21,26,27]. In contrast, a recent study revealed that SIRT1 may be a detrimental factor in certain inflammatory circumstances [28]. EX-527 inhibits LPS-induced elevation of IL-6 and TNF-α in the plasma. In addition, EX-527 ameliorates LPS-induced histological changes in the lung tissue, which is accompanied with reduced myeloperoxidase content and inhibited the induction of tissue factor and plasminogen activator inhibitor-1. Therefore, selective inhibition of SIRT1 by EX-527 might improve endotoxemia-related acute lung injury moderately via inhibition of mTOR, which suggests that selective SIRT1 inhibitors may have potential for the pharmacological treatment of inflammatory lung injury [28].

Although SIRT1 plays a pivotal role in the prevention of HFD-induced liver fibrosis development, the functional role of SIRT4 in the progression of hepatic fibrosis remains unknown. In this study, we explored the effect of EX-527, a SIRT1-selective inhibitor, on the progression of high-fat diet (HFD)-induced fatty liver or fibrosis in Zucker diabetic fatty (ZDF) rats. A previous study indicated that EX-527 protected against HFD-fed diabetic nephropathy in ZDF rats [29]. However, the actual mechanism underlying the effect of a SIRT inhibitor in the progression of HFD-induced hepatic fibrosis is unclear. We hypothesized that EX-527 protects against the progression of liver fibrosis in HFD-induced ZDF rats by modulating blood glucose, oxidative stress, and the expression of inflammatory cytokines.

## 2. Materials and Methods

### 2.1. Chemicals and Materials

EX-527 (6-chloro-2,3,4,9-tetrahydro-1H-carbazole-1carboxamide) was procured from Sigma–Aldrich Biotechnology (St. Louis, MO, USA). ELISA kits for the analysis of alanine aminotransferase (ALT), aspartate aminotransferase (AST), r-glutamyl transferase (r-GPT), and alkaline phosphatase (ALP) were procured from Abcam (Cambridge, UK). Assay kits for malondialdehyde (MDA), glutathione (GSH), catalase (CAT), and superoxide dismutase (SOD) were purchased from Cayman Chemical (Ann Arbor, MI, USA). Primary antibodies specific to liver fibrosis markers α-SMA, collagen 1, vimentin, Smad2/3, p-Smad2/3, Smad4, Smad7, p-PI3K, PI3K, p-Akt, Akt, p-PTEN, PTEN, Bcl-2, Bax, cleaved-caspase-3, p53, and β-actin were procured from Cell Signaling Technology (Danvers, MA, USA). Horseradish peroxidase (HRP)-conjugated secondary antibodies were acquired from Santa Cruz Biotechnology (Santa Cruz, CA, USA).

### 2.2. Experimental Design

**Figure 1:**
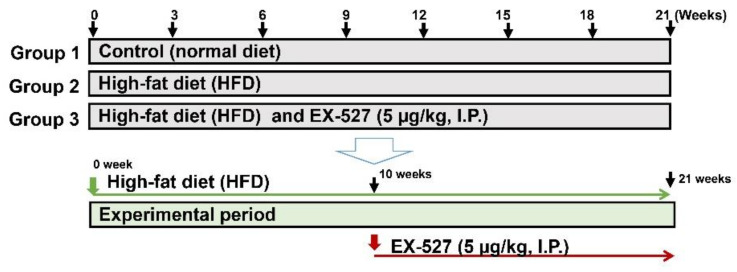
Experimental design. After 10 days of adaption, Zucker diabetic fatty (ZDF) rats were divided randomly into two groups: the normal diet (ND) group was fed a standard chow diet (*n* = 6) and the experimental group was fed a high-fat diet (HFD) (*n* = 12). After ten weeks of feeding the HFD, the rats were divided into two groups (*n* = 6/group) that were fed a HFD (*n* = 6) and a HFD followed by EX-527 administration (HFD+EX-527) for 21 weeks.

Male ZDF rats (body weight 300 ± 25 g) were procured from Central Lab Animal Inc. (Seoul, Korea). The rats were kept at normal temperature (24 ± 0.5 °C), relative humidity (54–58%), and a 12 h dark and light cycle, under specific pathogen proof conditions. The rats were acclimated to the laboratory conditions for ten days before the start of the experiment. The Sungkyunkwan University Animal Ethics Committee (SKKU-2018-10-32-1) approved the experimental methodology. The HFD, comprising carbohydrates, fats (60%), proteins, minerals, fiber, and vitamins was obtained from Research Diets, Inc. (New Brunswick, NJ, USA) and fed to the rats for 11 weeks to induce diabetes. EX-527 (5 μg/kg, twice weekly) was administered intraperitoneally (i.p.) to HFD-fed rats for ten weeks [29,30]. The normal diet-fed rats received diets which were devoid of fats. Glucose levels were determined by using a glucometer (ACCU-CHEK; Daeil Pharm. Co Ltd., Seoul, Korea). A rat with a glucose level of more than 300 mg/dL was considered as diabetic and used for further study. All the experimental ZDF rats were randomly distributed into three groups (*n* = 6). Rats were anesthetized after 21 weeks of treatment. The abdominal vein was used for blood collection and transferred into heparinized tubes. Serum was obtained following the centrifugation of blood at 2000× *g* for 10 min and transferred immediately at −80 °C for storage until further analysis. The major organs (liver) were collected and perfused with saline and stored at −80 °C for further analysis, as shown in Figure 1.

### 2.3. Serum Biochemical Analysis

Serum was collected into sterile tubes and frozen at −80 °C within 2 h of collection until use. AST, ALT, ALP, and r-GPT were evaluated using a VetScan analyzer (Abaxis, Inc., Union City, CA, USA). Total cholesterol (TC) was analyzed by a spectrophotometer at 560 nm. Low-density lipoprotein (LDL), triglycerides (TG) and high-density lipoprotein cholesterol (HDL-C) were estimated using a UV-visible spectrophotometer (JASCO, V-650, Japan) at 505 nm.

### 2.4. Histopathological Examination and Masson’s Trichrome Staining

Paraffin-embedded specimens were sectioned at 3–5 µm. Sections were fixed in 10% neutral buffered formalin overnight and then dehydrated with 70% ethanol. To detect the morphological alteration in liver tissue, sections were stained with hematoxylin & eosin (H&E) or Masson’s trichrome (MT) stain. Collagen deposition and degree of fibrosis were investigated using MT staining. A light microscope at 200× magnification (Zeiss Axiophot, Oberkochen, Germany) was used for capturing the photomicrographs.

### 2.5. GSH Content Determination

The content of glutathione (GSH) was estimated by using a commercially available kit (Cayman Chemical., Ann Arbor, MI, USA), in accordance with manufacturer’s protocol. Liver samples (100 mg) were homogenized with 5% metaphosphoric acid and centrifuged for 12 min at 12,000× *g*. The supernatant was collected following centrifugation and stored at 4 °C. Finally, the reaction mixture was detected at 405 nm absorbance by Vet Scan analyzer (Abaxis, Inc., Union City, CA, USA). The content of GSH is expressed as pmol/mg protein. The level of oxidized GSH (GSSG) in the liver tissue were measured using Ellman’s reagent-based assay with commercially available kits (Cayman Chemicals Company, Ann Arbor, MI, USA).

### 2.6. Analysis of SOD Activity

Superoxide dismutase (SOD) catalyzes the conversion of superoxide anion into H_2_O_2_ and O_2_, and its activity was estimated using a commercial kit (Cayman Chemical Co, Ann Arbor, MI, USA) according to the manufacturer’s protocol. The liver tissue (100 mg) was mixed gently in ice cold 5% (*w*/*v*) metaphosphoric acid and centrifuged at 1500× *g* for 5 min. The diluted radical detector samples (200 µL) were mixed with 10 µL supernatant. Twenty microliters xanthine oxidase was added and the absorbance at 440 nm was analyzed. SOD activity is stated as U/mg protein.

### 2.7. Assay of CAT Activity

Catalase (CAT) activity determination was based on the enzymatic reaction with methanol in the presence of hydrogen peroxide, a toxic byproduct of pathogenic reactive oxygen species (ROS) production and normal aerobic metabolism. CAT activity was estimated using a colorimetric assay kit (Cayman Chemical Co.) according to the manufacturer’s protocol. Liver samples (100 mg) were homogenized with cold buffer (pH 7) and centrifuged for 12 min at 10,000× *g* at 4 °C. The supernatant was collected following centrifugation and kept at 4 °C. Finally, the reaction mixture was added to tissue samples, and the absorbance was recorded at 570 nm. CAT activity is specified as nmol/mg protein.

### 2.8. Assay of MDA

The content of malondialdehyde (MDA) was estimated using a colorimetric assay kit as per the manufacturer’s protocol. MDA was analyzed in the form of thiobarbituric acid substances. Equal volumes (100 μL) of sodium dodecyl sulfate and sample were mixed in a 5 mL conical vial. The mixture was added to 0.4 mL of 1% thiobarbituric acid in 0.2 mL (20%) H_3_PO_4_ and 50 mm NaOH and was mixed gently. The mixture was incubated on ice before being heated for 15 min at 100 °C. The samples were then centrifuged for 10 min at 1600× *g*. Finally, the absorbance of the samples was read at 540 nm.

### 2.9. Assay of ROS

Reactive oxygen species (*ROS)* was analyzed using a commercial assay kit (Cell Biolabs, San Diego, CA, USA) as per the manufacturer’s protocol. At the beginning of the ROS assay, all the standard and tested samples were transferred into individual well. For fluorescence measurement, 50 µL of unknown sample or hydrogen peroxide standard were added into the well. Then, the tested as well as standard samples were mixed and Incubated for 5 min. After that, 100 µL of DCFH solution was added to each well and the plate was incubated for 15–45 min at room temperature. Finally, fluorescence was measured at 480 nm excitation / 530 nm emission.

### 2.10. Assay of Proinflammatory Cytokines

The inflammatory cytokine concentrations were investigated using ELISA kits (Abcam, Cambridge, MA, USA) as per the manufacturers’ protocol. The HFD induced-liver fibrosis rats showed a significant increase in IL-6, IL-1β, and TNF-α concentrations, in serum. IL-6- Standard, control and tested samples were transferred into wells of IL-6 specific antibody coated plate. The biotinylated specific antibody for standard and samples for IL-6 were incubated at room temperature. After that, HRP conjugated streptavidin was transferred in each well followed by washing. After a certain incubation, the TMB solution was added to each well. Finally stop solution was added to each well. The absorbance was detected by spectrophotometer at 450 nm. IL-1β, and TNF-α was also detected by the similar method as mentioned in IL-6. The concentrations of IL-6, IL-1β, and TNF-α were determined from standard plots.

### 2.11. Western Blot Analysis

Liver tissues (50 mg) were homogenized using RIPA lysis buffer (50 mg: 1 mL) and centrifuged for 12 min at 12,500× *g*. Protein concentrations were estimated using the BCA protein assay kit (Thermo Scientific, Pierce, MA, USA). Equal amounts of protein were denatured by boiling with the sample buffer for 5 min.

The sample buffer was prepared by 3 ml of 1M Tris-HCl, bromophenol blue 0.05 g and 10 mL of 10% SDS were mixed with 12.5 mL of glycerol, and finally 24.5 mL of distilled water was added. The total volume was made up to 50 mL. The storage condition of sample buffer was −20 °C. The protein samples were subjected to 6–12% sodium dodecyl sulfate-polyacrylamide gel electrophoresis. The gels were then transferred to polyvinylidene difluoride (PVDF) membranes (Millipore, Burlington, MA, USA). The membranes were incubated in TBS-T (50 MM Tris–HCl, pH 7.6, 0.1% Tween-20 and 200 mM NaCl) for 60 min with 5% skimmed milk and incubated with specific primary antibodies (1:1000) against Bcl-2, Bax, Cleaved caspase-3, p53, α-SMA, collagen I, vimentin, Akt, p-Akt, p-PTEN, PTEN, TGF-β1, Smad2/3, p-Smad2/3, Smad4, SIRT1, SIRT2, SIRT3, SIRT4, and β-actin for 12 h at 4 °C. Then the membranes were washed with TNT buffer for 60 min, incubated with a secondary antibody conjugated with HRP, such as anti-rabbit immunoglobulin G (1:10,000) or anti-mouse immunoglobulin G (IgG, 1:10,000), for 30 min, and then washed again with TNT buffer for 60 min. The TNT buffer was prepared by adding 700 mL of distilled water, 200 mL of methanol and 100 mL of 10× TG. The total volume was made up to 1000 mL. Finally, the protein expression and band intensity were detected using an enhanced chemiluminescence (ECL)-plus kit (Amersham Biosciences, Little Chalfont, UK).

### 2.12. mRNA Expression Analyses

Total RNAs were extracted (from the liver) using Trizol reagent (ref. no. 15596018, Life Technologies, Carlsbad, CA, USA). RNA was quantified using nanodrop followed by cDNA synthesis using Maxime RT PreMix (cat. no. 25081/96, Intron Biotechnology, Seoul, Korea) containing oligo-dT primer according to the manufacturer’s instruction. Synthesized cDNA was used as template for PCR amplification. The reaction was performed in 20 μL volume consisting of DNA polymerase (cat. no. 501-025, GeneAll, Seoul, Korea) at 95 °C for 3 min for an initial denaturation followed by 35 cycles at 95 °C for 30 s, 55 °C for 30 s, and 72 °C for 30 s, and a final extension at 72 °C for 5 min. Beta-actin gene was used as reference control in the reverse transcription-polymerase chain reaction (RT-PCR) experiment. 

### 2.13. Analysis of TGF-β1

The content of transforming growth factor beta (TGF-β1) in the serum was estimated using an ELISA kit (R&D Systems, Minneapolis, MN, USA) in accordance with the manufacturer’s directions. Before starting the experiment, the samples were activated with of 2.5 N acetic acid/10 M urea. In order to avoid interference from the matrix, a dilution ratio of 1:24 was used for the samples. The diluted samples were transferred to wells coated with a specific monoclonal antibody for TGF-β1 and incubated for 1 h. The plate was washed and mixed with 100 μL of TGF-β1 conjugate which was added onto the plate and incubated at room temperature for 2 h. Then the substrate solution was transferred to individual wells followed by washing and incubated for 30 min at room temperature. Finally, the absorbance of the samples was measured at 450 nm.

### 2.14. Hydroxyproline Analysis

The total amount of collagen was measured by the hydroxyproline assay. The assay was performed using an ELISA kit (Cell Biolabs) in accordance with the manufacturer’s protocol. Liver tissues (100 mg) were homogenized with 10 mL of 5 N HCl for hydrolysis and incubated overnight at 120 °C. After incubation, the hydrolyzed product was passed through a 0.45 µm PVDF syringe filter (Sigma–Aldrich, St. Louis, MO, USA). It was left to dry under vacuum for 45 min at 70 °C. All samples were analyzed in duplicates, and the mean values were used for analysis. The hydroxyproline level is specified as micrograms per milligrams of protein.

### 2.15. Immunohistochemical Assay

Immunohistochemical assay was performed to investigate the expression of SIRT1, SIRT2, SIRT4, α-SMA, and TGF-β. The slides were transferred to a xylene chamber and different grade of pure alcohol series and were transferred to 3% H_2_O_2_ to quench the peroxidase activity. A 4% BSA solution was used for blocking and incubated at 37 °C for 1 h, followed by three washes with tris-buffered saline (TBS) and interaction with the primary antibodies against SIRT1 (1:1000), SIRT2 (1:500), SIRT4 (1:500), α-SMA (1:200), and TGF-β (1:300) at 4 °C overnight. The sections were washed twice with TBS followed by incubation with the secondary antibody, conjugated with HRP for 45 min at room temperature. Diaminobenzidine tetrahydrochloride (DAB) was used as a visualizing agent. Microscopy was used to identify histological changes.

### 2.16. TUNEL Assay

The TUNEL assay was performed to investigate the fragmentation of DNA during apoptosis. DeadEnd^TM^ colorimetric system (Promega, Madison, WI, USA) was employed to identify apoptotic cells in the liver tissue of diabetic rats.

### 2.17. Statistical Analysis

Data are expressed as mean ± S.D. of six animals. The level of statistical significance was determined using one-way analysis of variance (ANOVA), followed by Tukey’s HSD post hoc test for multiple comparisons. Statistical analyses were performed using GraphPad Prism v5.0 (GraphPad Software, San Diego, CA, USA). *p* < 0.05 indicated statistical significance.

## 3. Results

### 3.1. Effect of SIRT4 Expression in Human Liver Fibrotic Tissues

**Figure 2:**
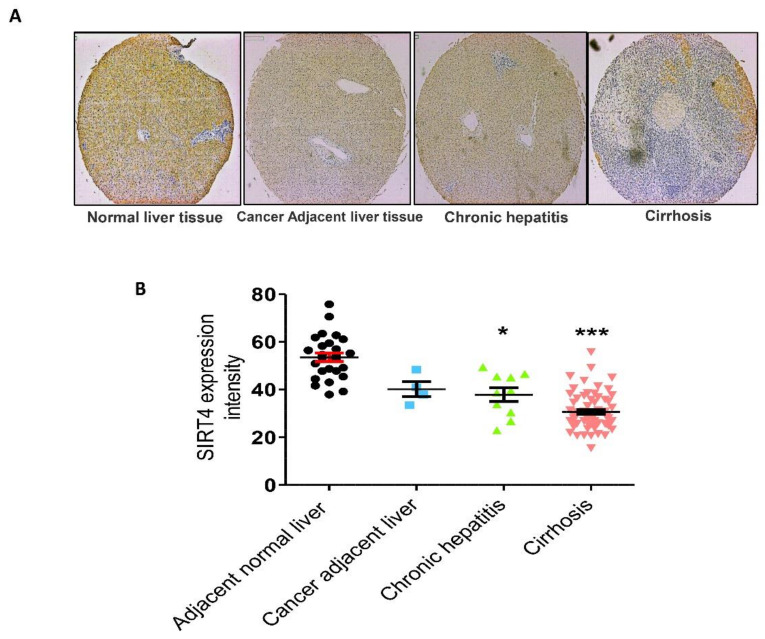
Expression of SIRT4. (**A**) SIRT4 protein was immunostained with a specific antibody in normal human liver tissue, cancer adjacent liver tissue, and chronic hepatitis and cirrhosis tissue samples and observed under microscopy at 400× magnification. In comparison with normal liver tissues, chronic hepatitis and cirrhosis tissues exhibited lower expression levels of SIRT4. (**B**) Immunoreaction scoring of SIRT4 between human liver tissue samples (cancer adjacent liver tissue, chronic hepatitis and cirrhosis) and normal liver tissue samples. The values are mean ± S.D. Statistical analysis was performed using Kruskal–Wallis test for single comparisons. * *p* < 0.05, *** *p* < 0.001. The cancer adjacent liver, chronic hepatitis and cirrhosis were compared with the adjacent normal liver.

Previously, it was reported that the expression of SIRT4 mRNA and protein is increased in patients with NAFLD, which suggested that SIRT4 might promote the progress of NAFLD [21]. However, the direct correlation between SIRT4 expression and hepatic fibrosis was unknown. In our study, we examined whether SIRT4 is overexpressed in human hepatic fibrosis tissues. We found that SIRT4 expression is significantly reduced in human liver specimens from patients with fibrosis in contrast with that in normal tissues, as shown in Figure 2. 

### 3.2. Effects of EX-527 on Body Weight, Liver Weight, and Blood Glucose in HFD-Fed Rats

**Figure 3:**
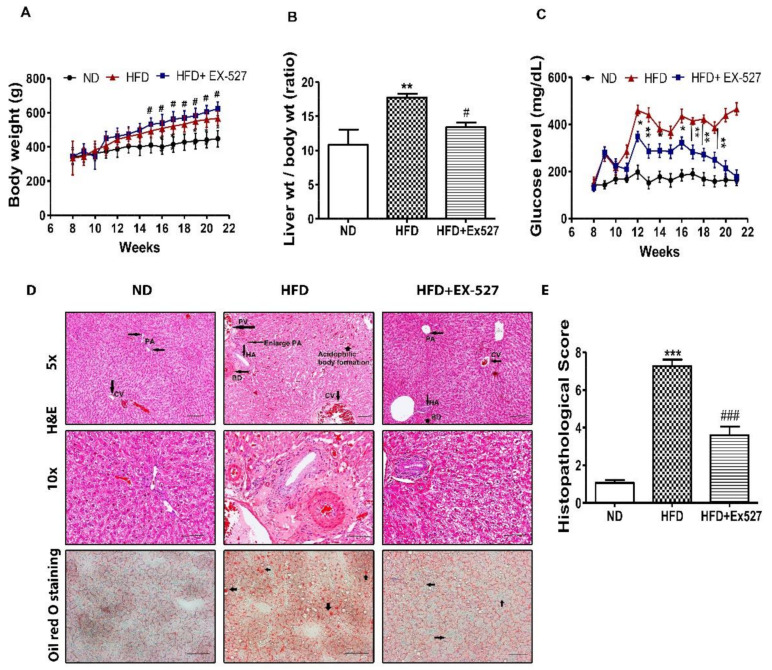
In vivo effects of EX-527 on glycemic status and microscopic appearances of the liver in HFD-induced diabetic rats. Male ZDF rats were fed either control diet or a high-fat diet (HFD) for 11 weeks. (**A**) HFD-induced body weight gain in ZDF rats. A significant increase in body weight gain was observed in EX-527-treated HFD-fed rats. (**B**) Effect of EX-527 on relative liver weights. (**C**) Effect of EX-527 on fasting blood glucose concentration in HFD-fed rats (mg/dL). (**D**-Upper) Representative histopathological analysis of hematoxylin and eosin (H&E)-stained liver sections were performed from the experimental group rats. HFD-induced diabetic rats at 21 weeks showed abnormalities associated with bile duct hyperplasia, which was induced by high fat diet. It would show inflammation in or surrounding the proliferated ducts (focal proliferation of ducts). HFD-fed rat tissues showed an increase in fibrous tissue thickness, an expansion of the portal area and central veins, and an increase in hepatic artery. Light micrographs of experimental group rats (ND, HFD and HFD+ EX-527). CV, central vein; PV, portal vein; PA, portal area; HA, hepatic artery; BD, bile duct. Images are representative of three animals per experimental group (magnification × 200; bar = 50 μm. (**D**-lower) Effect of EX-527 on the liver tissue of HFD-induced diabetic rats evaluated by oil red-O staining. HFD-induced diabetic rats at 21 weeks showed the liver tissue sections stained with oil red O showed a large number of red lipid droplets, and an increase in abnormal lipid storage indicating the presence of steatosis in the liver. The arrowhead shows the lumen of a blood vessel. Images are representative of three animals per group (magnification × 200; bar = 100 μm. (**E**) Histological score of H&E staining of liver section. The values are mean ± S.D. of six rats per group. Statistical analysis was performed using one-way analysis of variance (ANOVA) followed by Tukey’s honestly significant difference (HSD) post hoc test for multiple comparisons. * *p* < 0.05, ** *p* < 0.01, and *** *p* < 0.001 compared with the normal diet group (ND); ^#^ *p* < 0.05, ^###^ *p* < 0.001 compared with the HFD group.

Food intake (g/body weight) of the individual group was not altered (data not shown). Prolonged feeding of HFD resulted in severe morphological alterations of the liver in rats. However, management with EX-527 mitigated morphological alteration of the liver. Body weight gain was significantly reduced in HFD-fed rats compared with EX-527 treated rats. However, administration of EX-527 in HFD-fed rats increased the body weight in HFD-fed rats, as shown in Figure 3A. A significant increase in liver weight was observed in HFD-fed rats in contrast with normal diet-fed rats, while EX-527 markedly reduced HFD-induced liver weight gain, as displayed in Figure 3B. This suggested that EX-527 might prevent liver injury in HFD-induced rats. In HFD-fed rats, the fasting serum glucose concentration was significantly augmented compared with that in normal diet-fed rats. Two weeks after HFD-feeding, blood glucose concentration was increased significantly in HFD fed rats than that of normal diet-fed rats. However, fasting blood glucose level was dramatically decreased in the HFD-fed rats following EX-527 treatment, as shown in Figure 3C.

### 3.3. Effect of EX-527 on Liver Histological Analysis in HFD-Induced Diabetic Rats

Histopathological analysis clearly indicated normal cell morphology of the portal area and the central vein in the liver of normal diet-fed rats. However, livers of HFD-fed rats displayed severe damage around the connective tissue and the central vein. HFD induced bile duct hyperplasia showed inflammation in or surrounding the proliferated ducts (focal proliferation of ducts). Simple bile duct proliferation may remain static for some time, regress or progress to a more extensive form leading to the appearance of cholangiofibrosis. It seems liver bile duct hyperplasia, primary biliary fibrosis or cholangiofibrosis might be prominent in HFD. In treatment with EX-527, it was significantly reduced. In HFD, the spreading of bile duct was very clearly visible and many small strands in dark red were present, which might be sign of fibrosis. These changes were not noticed in treatment and ND groups. The inflammatory infiltrates were also noted in the liver of HFD-fed rats. However, such cell morphology abnormalities were not observed in the liver tissue of HFD-fed EX-527-treated rats, as shown in Figure 3D,E. HFD-fed rats had dramatically increased fat accumulation in the liver tissues detected using oil red-O staining. Normal diet-fed rats did not show any lipid droplets in the liver, whereas lipid droplets were diffuse, and granular accumulation was significantly increased in the livers of HFD-fed rats. EX-527-treated rats exhibited fewer lipid droplets in the liver, as shown in Figure 3D. These data suggest that EX-527 inhibits the development of hepatic fibrosis and injury in HFD-fed rats by reducing lipid accumulation in hepatocytes.

### 3.4. Effect of EX-527 on SIRT Expression in the Liver of HFD-Induced ZDF Rats

**Figure 4:**
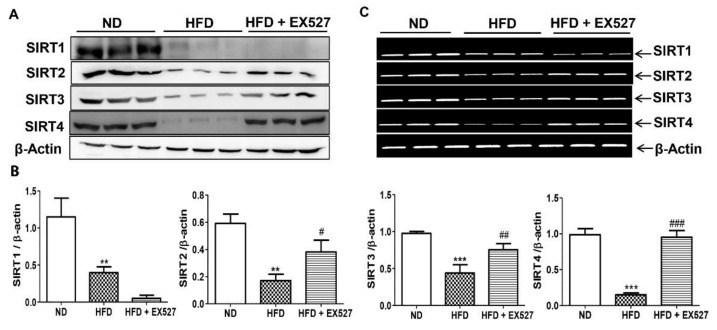

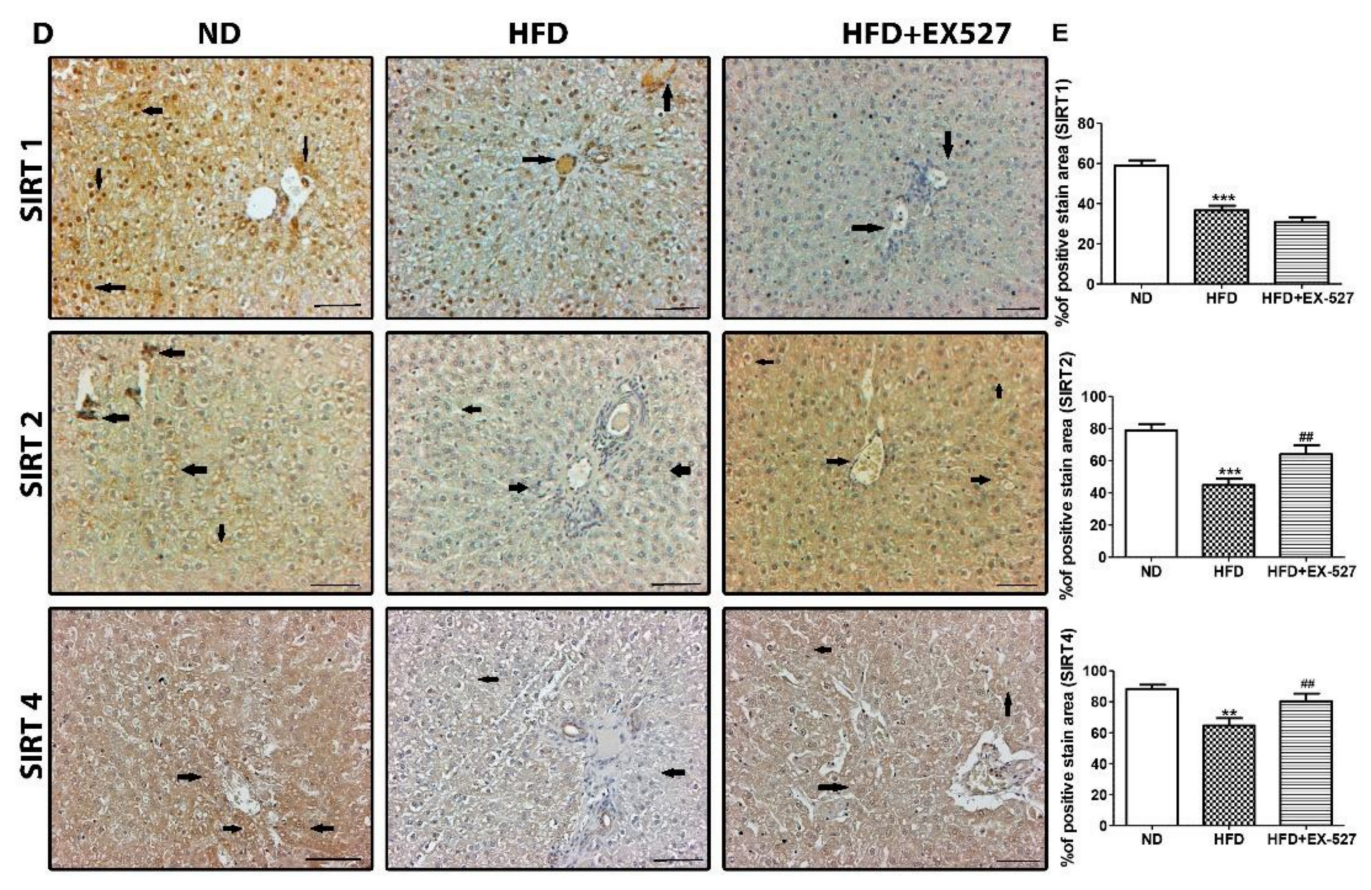
Effect of EX-527 on SIRT expression in the liver of HFD-induced diabetic rats was studied by Western blot, polymerase chain reaction (PCR) and immunohistochemistry. Male ZDF rats were fed either control diet or a high-fat diet (HFD) for 11 weeks. (**A**) Representative Immunoblotting bands of SIRT1, SIRT2, SIRT3 and SIRT4, were shown using an experimental model of HFD-induced diabetic rats. β-Actin expression was used as the loading control. (**B**) The intensity of the bands was analyzed densitometrically by ImageJ software. (**C**) mRNA expression levels of SIRTs (SIRT1, SIRT2, SIRT3 and SIRT4) was analyzed by PCR. (**D**) Representative Immunohistochemical staining of SIRT1 SIRT2 and SIRT4 were shown for in the liver tissue of HFD-induced diabetic rats. Black arrows represent expression rate. Original magnification: 200×, scale bar: 100 μm. ND, normal diet; HFD, high-fat diet. (**E**) Representative the percentage (%) of positive area staining of SIRT1 SIRT2 and SIRT4. The values are mean ± S.D. of six rats per group (*n* = 6). Statistical analysis was performed using one-way analysis of variance (ANOVA) followed by Tukey’s honestly significant difference (HSD) post-hoc test for multiple comparisons. ** *p* < 0.01, and *** *p* < 0.001 compared with the normal diet group (ND); ^#^ *p* < 0.05, ^##^ *p* < 0.01, and ^###^ *p* < 0.001 compared with the HFD group. ND, normal diet; HFD, high-fat diet.

On basis the histological indication of liver fibrosis in HFD-fed rats, we analyzed the expression of SIRTs in the liver tissues of HFD-fed diabetic rats. The expressions of SIRT1, SIRT2, SIRT3, and SIRT4 were significantly reduced in HFD-fed diabetic rats compared with that in normal diet-fed rats, whereas EX-527 administration upregulated SIRT2, SIRT3, and SIRT4 expression, as shown in Figure 4A,B. Additionally, mRNA levels of SIRTs were reduced in the livers of HFD-fed diabetic rats. However, the expressions of SIRT2, SIRT3, and SIRT4 were increased following the administration of EX-527 in HFD-fed rats, as shown in Figure 4C.

For further confirmation of the protective effects of EX-527 on liver fibrosis in HFD-fed diabetic rats, we performed immunohistochemistry analysis to measure SIRT expression in the liver tissues. The expression of SIRTs was increased in the liver of normal diet-fed rats, whereas SIRT1, SIRT2, and SIRT4 expressions were dramatically downregulated in the liver tissue of HFD-fed rats, as shown in Figure 4D,E. Additionally, the expression of SIRT1 was further reduced following EX-527 treatment, corroborating the Western blot analysis data. However, SIRT2, and SIRT4 levels were increased in HFD-fed rats following EX-527 treatment, as displayed in Figure 4D,E.

### 3.5. EX-527Attenuates HFD-Induced Hepatic Injury 

**Figure 5:**
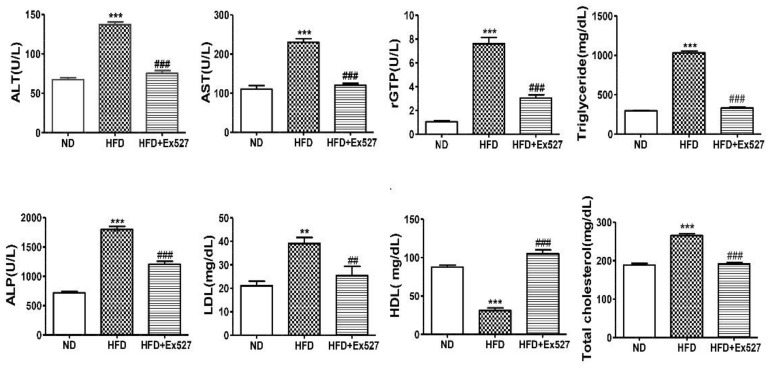
Effect of EX-527 on biochemical parameters in the serum of HFD-fed diabetic rats. Male ZDF rats were fed either control diet or a HFD for 11 weeks. Effect of EX-527 (5 μg/kg) on serum alanine aminotransferase (ALT), aspartate aminotransferase (AST), r-glutamyl transferase (r-GPT), triglycerides (TG), alkaline phosphatase (ALP), low-density lipoprotein (LDL), high-density lipoprotein (HDL), and total cholesterol (TC) in HFD-fed diabetic rats. The values are mean ± S.D. of six rats per group. Statistical analysis was performed using one-way ANOVA followed by Tukey’s HSD post-hoc test for multiple comparisons. ** *p* < 0.01, and *** *p* < 0.001 compared with the normal diet group (ND); ^##^ *p* < 0.01, and ^###^ *p* < 0.001 compared with the HFD group.

The serum levels of AST, ALT, ALP, r-GPT, LDL, TC, and TG were significantly increased in HFD-fed rats compared with those in normal diet-fed rats. However, the HFD-induced increase in these biochemical parameters was significantly inhibited by the administration of EX-527, as shown in Figure 5. The level of high-density lipoprotein (HDL) in HFD-fed rats was significantly increased after administration of EX-527. These data clearly indicate that EX-527 shows a protective effect in HFD-induced liver fibrosis, as displayed in Figure 5.

### 3.6. Effects of EX-527 on Hepatocyte Apoptosis in Diabetic Rats

**Figure 6:**
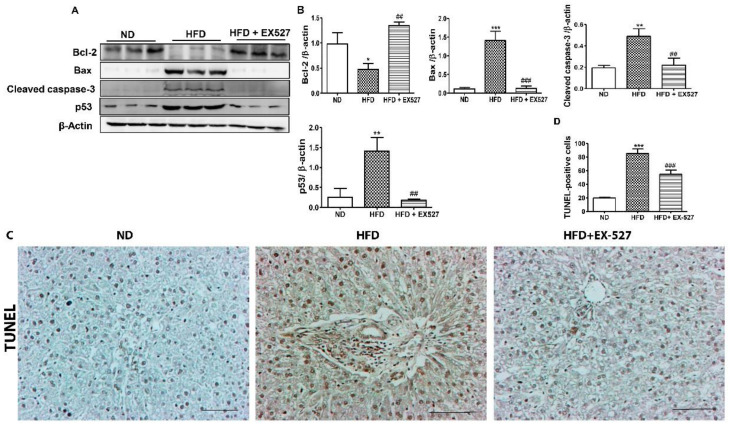
Effects of EX-527 on the expression of apoptosis-related proteins in the liver tissue of HFD-fed diabetic rats. Male ZDF rats were fed either control diet or a HFD for 11 weeks. (**A**) Representative Western blotting analysis of Bcl-2, Bax, cleaved caspase-3, and p53 in the liver tissue. β-Actin expression was used as the loading control. (**B**) The intensity of the bands was analyzed densitometrically by ImageJ software. (**C**) Apoptosis determined by TUNEL staining of the liver tissues of HFD-fed rats. Original magnification: 200×, scale bar: 100 μm. (**D**) Determined TUNEL-positive score index. The values are mean ± S.D. of six rats per group. Statistical analysis was performed using one-way ANOVA followed by Tukey’s HSD post-hoc test for multiple comparisons. * *p* < 0.05, ** *p* < 0.01, and *** *p* < 0.001 compared with the normal diet group (ND); ^##^ *p* < 0.01, and ^###^ *p* < 0.001 compared with the HFD group.

Bcl-2 and Bax are vital proteins that regulate apoptotic cell death. Our results revealed that Bcl-2 was significantly decreased and Bax, cleaved caspase-3, and p53 were upregulated in the liver of HFD-fed diabetic rats. However, these alterations in the liver of HFD-fed diabetic rats were significantly attenuated by EX-527, as shown in Figure 6A,B. This indicates that EX-527 has the potential to control liver fibrosis in HFD-fed diabetic rats. Next, we performed a TUNEL assay to measure chromatin condensation, which correlates with the degree of hepatic damage, as displayed in Figure 6C,D. Hepatocyte apoptosis was noticed in the liver of HFD-fed diabetic rats. Apoptotic cell death was markedly reduced following treatment with EX-527 in HFD-fed diabetic rats.

### 3.7. Effects of EX-527 on Hepatic Oxidative Stress Biomarkers in Diabetic Rats

**Figure 7:**
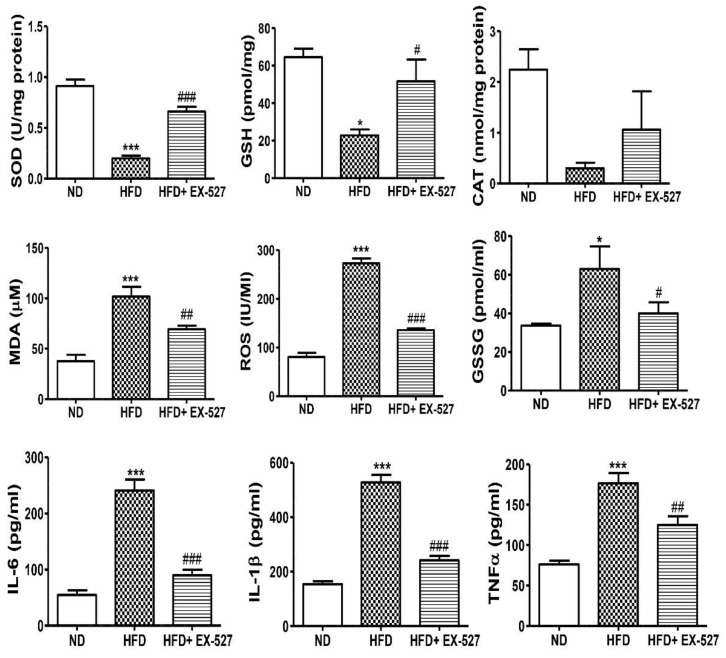
Effect of EX-527 on antioxidant components of the liver of HFD-fed diabetic rats. Male ZDF rats were fed either control diet or a HFD for 11 weeks. EX-527 (5 μg/kg, twice a week) was administered intraperitoneally (i.p.) for 10 weeks to HFD-fed rats. Changes in superoxide dismutase (SOD) activity, catalase (CAT) activity, glutathione (GSH) level, lipid peroxidation (MDA) level, reactive oxygen species (ROS) level and oxidized GSH (GSSG) were analyzed in the liver tissue of HFD-fed diabetic rats. Effect of EX-527 on the serum concentrations of proinflammatory cytokines in the rats with HFD-fed diabetes. Male ZDF rats were fed either control diet or an HFD for 11 weeks. The proinflammatory cytokines (IL-6, IL-1β, and TNF-α) were significantly reduced in EX-527-treated HFD-fed rats. The values are mean ± S.D. of duplicate experiments (six rats/group). Statistical analysis was performed using one-way ANOVA followed by Tukey’s HSD post hoc test for multiple comparisons. * *p* < 0.05, and *** *p* < 0.001 compared with the normal diet group (ND); ^#^ *p* < 0.05, ^##^ *p* < 0.01, and ^###^ *p* < 0.001 compared with the HFD group.

The antioxidant parameters, including GSH, SOD, CAT and MDA were investigated in HFD-fed rats. Additionally, the changes in the oxidative biomarkers such as GSSG and ROS were also measured. The GSH concentration was significantly reduced in the liver of HFD-fed rats compared with that in normal diet-fed rats. However, the concentration of GSH was significantly increased after treatment with EX-527. The higher level of GSSG was found in the liver of the HFD-fed rats in contrasted with the rats that were fed on a normal diet. However, the level of GSSG again decreased after treatment with EX527. The SOD activity was markedly decreased in HFD-fed rats whereas treatment with EX-527 recovered the SOD activity in HFD-fed rats to the normal level. Hepatotoxicity associated with the oxidative stress marker MDA is responsible for lipid peroxidation in the liver. Hepatic MDA content was significantly increased in HFD-fed diabetic rats in contrast with that in normal diet-fed rats. However, the MDA level was significantly decreased in rats treated with EX-527. CAT activity was decreased in the liver of HFD-fed rats compared with that in normal diet-fed rats, whereas administration of EX-527 increased CAT activity. Additionally, ROS assay was performed. A higher level of ROS was found in HFD-fed diabetic rats compared with that in normal diet-fed rats, whereas treatment with EX-527 significantly reduced ROS level, as shown in Figure 7.

### 3.8. EX-527 Inhibits the Inflammatory Cytokines in HFD-fed ZDF Rats

Inflammation is one of the important factors in the pathogenesis of fatty liver disease. The serum levels of proinflammatory cytokines IL-6, IL-1β, and TNF-α were analyzed and found to be increased 3–5-fold in HFD-fed rats; however, EX-527 treatment significantly decreased these levels, as shown in Figure 7. Our data illustrate the inhibitory role of EX-527 on proinflammatory cytokines, which may be associated with the upregulation of SIRT4.

### 3.9. Effect of EX-527 on the Production of ECM Proteins in the Liver

**Figure 8:**
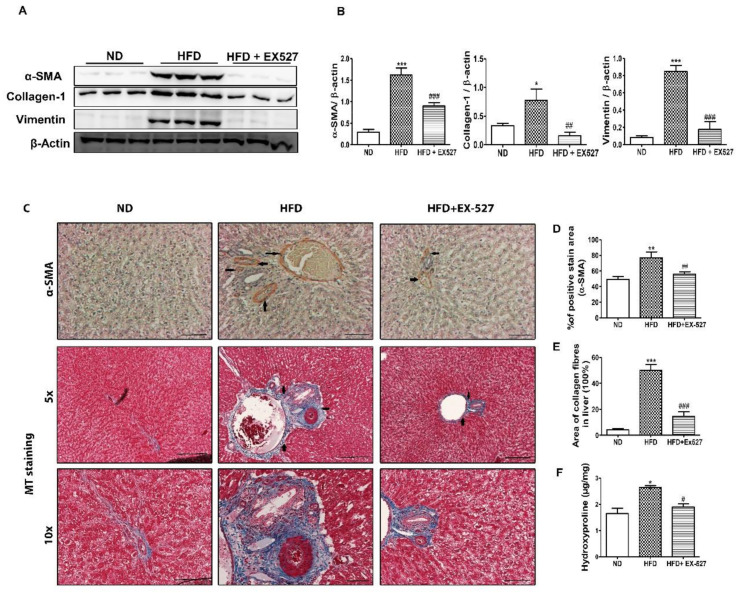
Effect of EX-527 on liver fibrous biomarkers in the liver of HFD-induced diabetic rats. (**A**) The expression of α-SMA, collagen-1 and vimentin in liver tissue was measured by Western blotting analysis, using an experimental model of HFD-induced diabetic rats. β-Actin expression was used as the loading control. (**B**) The intensity of the bands was analyzed densitometrically by ImageJ software. (**C**) Representative immunohistochemical staining of α-SMA in the liver of HFD-induced diabetic rats. Black arrows represent α-SMA expression and Masson’s trichrome (MT) staining. Black arrows represent collagen accumulation (blue color). Original magnification: 200×, scale bar: 100 μm. (**D**) Percentage (%) of positive area staining of α-SMA. (**E**) Percentage area (%) of collagen fibers in liver. (**F**) 4-hydroxyproline concentration in the liver of HFD-induced diabetic rats. Statistical analysis was performed using one-way ANOVA followed by Tukey’s HSD post hoc test for multiple comparisons. * *p* < 0.05, ** *p* < 0.01, and *** *p* < 0.001 compared with the normal diet group (ND); ^#^ *p* < 0.05, ^##^ *p* < 0.01, and ^###^ *p* < 0.001 compared with the HFD group.

To explore the pathogenesis of HFD-fed diabetic fibrosis in the liver, molecular markers of liver fibrosis were investigated using Western blotting. The protein expression of fibrosis markers such as α-SMA, collagen-1, and vimentin in the liver of HFD-fed rats was significantly higher than the normal diet-fed rats. However, the elevation in the expression of ECM proteins was significantly downregulated following treatment of EX-527 in HFD-fed diabetic rats, as shown in Figure 8A,B.

Immunohistochemical staining of the liver tissue was performed to detect α-SMA-positive cells in the liver tissue. The results indicate a higher expression rate of α-SMA in the fibrous septa and sinusoid in the livers of HFD-fed diabetic rats. However, administration with EX-527 significantly reduced the expression of α-SMA. This indicates that ECM production associated with HFD is significantly inhibited by EX-527, which restricts the development of liver fibrosis in diabetic rats, as shown in Figure 8C,D. Collagen accumulation in the liver is directly responsible for liver fibrosis. We performed MT staining to notice collagen deposition in the liver tissue. The results showed a higher collagen accumulation in the livers of HFD-fed diabetic rats accompanied by nodule formation. However, administration of EX-527 significantly suppressed the accumulation of collagen in the liver of HFD-induced diabetic rats. Collagen content, stained blue, was increased in the portal area of the liver of HFD-fed diabetic rats. This change was reversed by treatment with EX-527, as shown in Figure 8C,E.

Hydroxyproline is another vital parameter used to detect the severity of fibrosis in the liver [31]. Hydroxyproline was estimated by HPLC. A higher level of hydroxyproline was found in the liver of HFD-fed diabetic rats than that in normal diet-fed rats. However, hydroxyproline content in HFD-fed rats was significantly decreased after treatment with EX-527, as shown in Figure 8F.

### 3.10. EX-527 Restrored p-Akt and p-PTEN Expression in the Liver of HFD-Fed Rats

**Figure 9:**
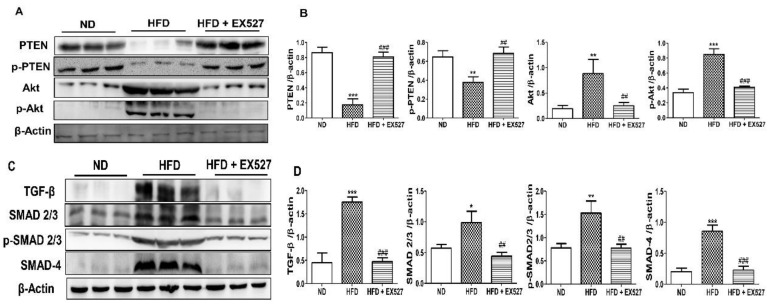

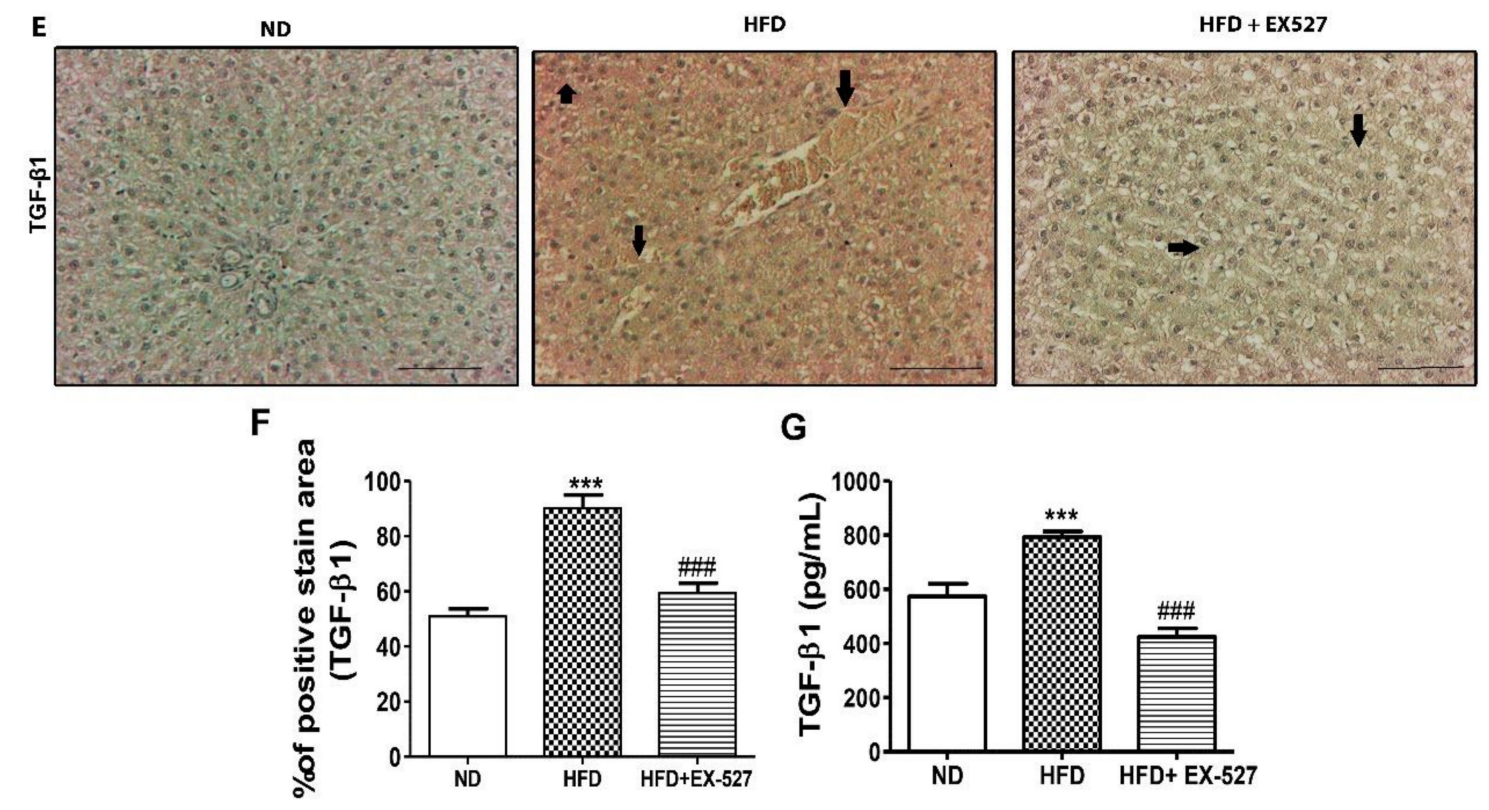
Effects of EX-527 on the expression of p-Akt and p-PTEN in the liver of HFD-fed rats. (**A**) Protein expression of tensin homolog (PTEN), p-PTEN, protein kinase B (Akt), and p-Akt was measured by Western blot analysis using an experimental model of HFD-induced hepatic fibrosis. β-Actin expression was used as the loading control. (**B**) The intensity of the bands was analyzed densitometrically by ImageJ software. Effect of EX-527 on TGF-β/Smads-related proteins in the liver of HFD-induced diabetic rats by Western blotting analysis. (**C**) Expression of TGF-β1, Smad2/3, p-Smad2/3, and Smad4 was measured by Western blot analysis in the liver tissue of HFD-induced diabetic rats. β-Actin expression was used as the loading control. (**D**) The intensity of the bands was analyzed densitometrically by ImageJ software. (**E**) Representative Immunohistochemical staining of TGF-β1 in the liver of HFD-fed diabetic rats. Black arrows represent TGF-β1 expression. Original magnification: 200×, scale bar: 100 μm. The Western blot results are representative of three separate experiments. (**F**) The positive area of TGF-β1 staining in the liver (%). (**G**) ELISA results of TGF-β concentration in the serum of HFD-induced diabetic rats. Statistical analysis was performed using one-way ANOVA followed by Tukey’s HSD post hoc test for multiple comparisons. * *p* < 0.05, ** *p* < 0.01, and *** *p* < 0.001 compared with the normal diet group (ND). ^##^ *p* < 0.01, and ^###^ *p* < 0.001 compared with the HFD group.

We explored the effect of TGF-β/Smad and Akt pathways in the development of liver fibrosis in HFD-fed diabetic rats. The results revealed that p-Akt protein expression was markedly increased in HFD-fed diabetic rats compared with that in normal diet-fed rats; however, the expression reduced following treatment with EX-527. This outcome was inversely correlated with PTEN protein expression. p-PTEN expression decreased in the liver of HFD-fed rats. However, the abnormal protein expression was restored following EX-527 treatment, as shown in Figure 9A,B.

### 3.11. TGF-β/Smad Signaling-Mediated Effects of EX-527 in HFD-Induced Liver Fibrosis

TGF-β is a vital parameter responsible for liver fibrosis via ECM regulation [32,33]. TGF-β expression in the liver was measured by Western blot analysis. The protein expression of HFD rats was higher in the liver of the HFD-fed diabetic rats than in the liver of the normal diet-fed rats. However, the protein expression was reduced after treatment with EX-527 in HFD-fed rats. Smad2/3 has a vital role in the TGF-β signaling pathway. The development of liver fibrosis depends on the phosphorylation of Smad2/3. The inhibition of phosphorylation of Smad2/3 and Smad4 prevents the downstream signaling pathway, which is responsible for liver fibrosis [34]. p-Smad2/3 and Smad4 expression was higher in the liver of HFD-fed diabetic rats than in the liver of normal diet-fed rats, whereas rats treated with EX-527 had a lower expression, as shown in Figure 9C,D. We measured TGF-β expression using immunohistochemistry analysis for confirmation of the protective effect of EX-527 in diabetic rats. The expression of TGF-β in the fibrotic liver tissue was significantly higher in HFD-fed diabetic rats than in the liver of the rats fed on a normal diet. However, treatment with EX-527 decreased TGF-β expression in HFD rats, as shown in Figure 9E,F.

Additionally, we also measured TGF-β concentration in the serum for confirmation of the protective effect of EX-527 in HFD-fed liver fibrosis rats. The TGF-β level in the serum of HFD-fed diabetic rats was significantly higher than that in liver of rats fed on a normal diet. However, treatment with EX-527 reduced the TGF-β level in HFD-fed diabetic rats, as displayed in Figure 9G.

## 4. Discussion

Various therapeutic agents (synthetic and natural isolated molecules) are available for the attenuation and treatment of HFD-induced diabetic liver fibrosis. However, the mechanism and pathophysiology of liver fibrosis remain unclear; therefore, potential treatments are needed. Sirtuins are a group of NAD(+)-dependent deacetylases, which have an extensive role in both alcoholic and nonalcoholic fatty liver diseases associated with oxidative damage [21,24,25]. The beneficial effects of SIRT1 in controlling hepatic lipid metabolism, regulation of oxidative stress and inflammation via deacetylating of some transcription factor against development of hepatic fibrosis is well established [35]. However, the protective effect of SIRT1 inhibitor in hepatic steatosis and fibrosis and its underlying mechanism is not clearly understood. In this respect, a selective SIRT1 inhibitor EX-527, showed its inhibitory activity against fibroblast activation markers, which reflects the importance of EX-527 in the development process of antidiabetic nephropathy drugs [29,36].

In the current study, we revealed the protective effect of EX-527 on HFD-induced liver fibrosis in a diabetic rat model. Our findings show that a low dose of EX-527 could alleviate hepatic fibrosis in diabetic rats. HFD-induced diabetic liver fibrosis increased oxidative stress and inflammation in the liver tissues of the HFD-fed diabetic rats in comparison to the rats that were fed a normal diet, as shown in Figure 7. These observations showed that this model is suitable for exploring the protective effects of EX-527 against hepatic fibrosis. Furthermore, body weight loss is a common phenomenon in the diabetic model. In the case of diabetes, inadequate insulin protects the physiology from receiving glucose from the blood into the body’s cells for supply energy. This physiology is competent for burning fat and muscle for the production of energy, resulting reduction of bodyweight. Another important reason behind the weight loss is excessive dehydration due to high blood glucose content [37,38]. In our study, body weight decreased, and liver weight increased in the HFD-fed diabetic rats; these are the distinctive features of HFD-induced liver toxicity. 

Our results reveal that serum enzymatic (AST, ALT, ALP, and rGTP) activities increased in the blood of HFD-fed rats compared with normal diet-fed rats. However, administration of EX-527 decreased the levels of hepatic damage biomarkers including AST ALT, ALP, and rGTP, as shown in Figure 5. These data clearly indicate that EX-527 effectively abolishes the elevation of the critical regulators of hepatic damage, whereas protective effects against hepatic fibrosis through SIRT1 expression were not influenced by EX-527 administration. It showed that EX-527 relieves HFD-induced liver injury by decreasing liver metabolic enzyme activity. This study highlights the possible hepatoprotective potential of EX-527, by pathway inhibition that alleviates liver damage in HFD-fed rats.

The mechanism of hepatic fibrosis in diabetes is complicated and depends on many factors. A pathophysiological approach associated with hepatic fibrosis is represented by a “two-hit” hypothesis [39]. Accumulation of TG in the liver is a primary criterion and is therefore the “first hit” in the progression of liver fibrosis in type 2 diabetes. This leads to the excessive generation of free radicals and lipid peroxidation in hepatocytes, whereas, the “second hit” signifies oxidative stress in the liver. The oxidative stress results not only in impairment of mitochondrial function and structure, but also leads to hepatic fibrosis, apoptosis, and nonalcoholic steatohepatitis [39,40]. Our result reveals that EX-527 restores the abnormalities associated with hepatic fibrosis, such as hyperlipidemia, decreases TG, and improves hepatic steatosis in HFD-induced diabetic rats. Liver fibrosis associated with diabetes (type 2) is identified by high levels of glucose in blood, TG, and very low-density lipoprotein. However, oxidative stress, lipid peroxidase, fibrogenic factors, and hepatic stellate cell proliferation are important factors for the development of liver fibrosis [41,42,43,44]. In this perspective, hematopoietic stem cells (HSCs) remain in an inactive state in hepatocyte. The activated HSCs are ready to proliferate and secrete α-SMA in specific circumstances, so that ECM is accumulated in the liver and induces hepatic fibrosis [45]. Our current study reveals that EX-527 could significantly decrease α-SMA and ECM levels in the liver of HFD-induced diabetic rats. Administration of EX-527 significantly decreases the level of blood lipid parameters including TC and TG, as shown in Figure 5.

The higher accumulation of TG in hepatocyte is the cause of hepatic fibrosis which leads to excess free radical generation and lipid peroxidation in the liver. Therefore, oxidative stress is one of the key factors of liver fibrosis. The molecular mechanism of hepatotoxicity is believed to be oxidative stress [46]. Previous reports demonstrate that ROS is responsible for liver fibrosis including in the epithelium and microvascular endothelium. The major antioxidative enzymes, such as MDA, glutathione (GSH), SOD and CAT, are all indicators of oxidative stress [47]. Currently, various reports have shown the protective effects of novel antioxidants against liver fibrosis [48,49]. Our study showed that CAT, SOD and GSH decreased in HFD-fed diabetic rats in comparison to normal diet-fed rats, as shown in Figure 7. However, the antioxidant activities were restored following treatment with EX-527. Therefore, EX-527 should be considered as a good antioxidant agent and plays a significant role in liver fibrosis. MDA is an indicator of liver peroxidation [49]. A higher level of MDA was found in the livers of HFD-induced diabetic rats, whereas, treatment with EX-527 significantly declined the MDA level, as shown in Figure 7. MDA is responsible for liver damage via ROS [46,47]. The ameliorative effect of EX-527 may be attributable to its free radical scavenging activity and antioxidant activity, which decreased oxidative stress due to HFD-mediated ROS generation. Inhibiting oxidative stress markers might explain the antifibrotic effect of EX-527 seen in diabetes-induced liver fibrosis.

Fibrogenesis is a late stage factor in chronic hepatic disorders. The hepatic stellate cells activation and extracellular matrix accumulation has a potential role in the development of fibrogenesis under circumstances of tenacious inflammation. The initial stage of hepatic injury is constantly associated with increased hepatocyte apoptosis. Therefore, hepatic cell death is the ultimate driver of the development of liver fibrosis and cirrhosis [50]. The alteration of Bax, Bcl-2 and cleaved caspase-3, results in hepatic mitochondrial damage [51]. We revealed whether EX527 inhibit the apoptosis pathway, by analyzing the protein expression of Bcl-2, Bax, and caspase-3 which are responsible for apoptotic cell death signaling and a crucial factor in mitochondrial damage. Current result showed that EX-527 significantly upregulated the Bcl-2 expression and inhibit the Bax, caspase-3 and p53 expressions. These effects demonstrate that EX-527 exerts the antiapoptotic capabilities to protect against liver injury.

Our results revealed that apoptotic protein expression was restored due to following treatment with EX-527. A higher level of injury cannot be repaired, and cells must be removed; p53 can induce apoptosis via a higher expression of BH3 (Bcl2 homologous 3) protein [52,53]. In addition, it was observed that the downregulation of p53, Bax and cleaved Caspase-3 modulates Bcl-2 expression facilitated cell death through Bcl-2/Bax signaling. Administration of EX-527 upregulates Bcl-2 expression and down-regulates p53, Bax and cleaved Caspase-3 in HFD-induced liver fibrosis rats. These results indicate that EX-527 might prevent liver fibrosis through its antiapoptotic potential.

Several mechanistic approaches are accountable for boosting collagen formation in the regulation of liver fibrosis. HSCs are a prime source of the extracellular matrix (ECM) proteins as well as fibrillar collagens which indirectly correlated with hepatic fibrosis [45]. The higher accumulation rate of ECM proteins is the indication of liver fibrosis. It is also considered that ECM proteins (e.g., collagen-1, α-SMA and vimentin) are only one of many potentially fibrogenic cell populations in hepatic disorder [54]. Based on these results, we propose that EX-527 is a potential candidate in liver fibrosis due to the inhibition of ECM. ECM proteins, such as collagen-1, α-SMA and vimentin, were significantly upregulated in the liver tissues of HFD-fed diabetic rats when compared with normal diet-fed rats. However, administration of EX-527 produced a downregulatory effect on the above-mentioned proteins. Similar to the immunohistochemistry analysis of α-SMA, the down-regulatory effect of ECM proteins by EX-527 in diabetic rats produced a protective effect against liver fibrosis. MT staining also showed that collagen expression was significantly increased in the HFD-fed diabetic rats compared with normal diet-fed rats, and the treatment of EX-527 decreased collagen accumulation in the liver.

Additionally, 4-hydroxyproline level was analyzed, which signifies the total collagen content in the hepatic tissue and is used as a signal for ECM accumulation [55]. The degree of fibrosis, as well as potential of antifibrotic agents, are determined by measuring 4-hydroxyproline level [53,54]. The level of hydroxyproline was markedly reduced in the hepatic tissues of EX-527-treated rats in comparison with HFD-induced diabetic rats. These results clearly suggest the antifibrotic potentiality of EX-527 against liver fibrosis of HFD-induced diabetic rats. The antifibrotic potential of EX-527 via the suppression of HSC stimulation and proliferation was validated using immunohistochemistry and MT staining.

ECM is driven mainly by TGF-β. The higher levels TGF-β is essential for myofibroblastic differentiation [54]. TGF-β1, a profibrotic cytokine, is an important factor in liver fibrosis. The levels of collagen I and collagen III were increased by TGF-β1. Various reports showed that TGF-β1 activates HSCs under oxidative stress conditions [56]. Therefore, we examined TGF-β1 expression to evaluate the protective effect of EX-527 in HFD-induced liver fibrosis rats. The results show that the expression of TGF-β1 is significantly upregulated in HFD-induced liver fibrosis rats compared with normal diet-fed rats, as shown in Figure 9C–E,G. The expression of TGF-β1 in the hepatic site was significantly decreased following treatment with EX-527. The down-regulatory effects of TGF-β1 might be associated with the antifibrotic capability of EX-527. The various cellular responses and signaling of TGF-β1 are triggered by the phosphorylation of Smad2/3 [57,58]. TGF-β1 stimulates ECM protein production through the activation of HSCs by the Smad pathway [59,60]. Results from this report reveal that EX-527 treatment decreases the expression of p-Smad 2/3 and Smad 4 in HFD-induced liver fibrosis rats, as displayed in Figure 9C,D.

Excessive oxidative stress and hepatic TG may lead to tissue damage and activation of repair pathways. These repair processes involve the recruitment of immune cells, angiogenesis, HSC activation, and subsequent ECM deposition. Activation of the MAPK pathway depends on TGF-β1 independently of Smad [61]. In this respect, p-Akt protein expression is significantly increased in the liver of HFD-induced liver fibrosis rats compared with normal diet-fed rats. The higher expression of p-Akt in the liver of HFD-induced diabetic rats is restored by the administration of EX-527 in fibrotic rats. Hence, the overall suggestion is that EX-527 inhibits the formation of EMT through the non-Smad or TGF-β1 pathway, and that TGF-β1 controls hepatic fibrosis through the Akt pathway, as shown in Figure 9.

The activation of proinflammatory cytokines promotes the development of chronic hepatitis [62,63]. 

The inhibition of IL-6, IL-1β, and TNF-α improve the hepatic aggravation in fibrogenesis. During hepatic injury, hepatic macrophages play a vital role in the fibrogenic process [64]. Additionally, TNF-related signaling induces immune cell recruitment and cytokine release, which is directly responsible for hepatic inflammation [65]. Current studies have revealed that the significant inhibitory effect of EX-527 on proinflammatory cytokines (IL-6, IL-1β, and TNF-α) might lead to a protective effect in hepatic fibrosis, as shown in Figure 7.

However, previous report revealed SIRT1 inhibition in fetal hepatocytes of humans in an increased lipid and glucose level intracellular [66]. Obesity is modulated by SIRT1. A significantly lower level of SIRT1 was found in obese associated liver steatosis compared to normal patients. [67]. Our previous study revealed that treatment with SIRT1 inhibitor attenuated the fibrosis in the kidney [29]. SIRT3 has been recognized as a vital parameter in caloric restriction related metabolic changes [68]. SIRT3 control the mitochondrial proteins functions which are involved in in oxidative phosphorylation and the antioxidant response system [69,70,71,72,73,74]. The acetylation levels of mitochondrial electron transport chain were controlled by SIRT3 which regulates ATP synthesis [75]. The levels of ATP were decreased by more than 50% in the liver of mice lacking SIRT3 [70]. Published reports revealed that obesity and chronic HFD decreased the SIRT3 expression. [76,77]. The enzymology-associated SIRT4 metabolism is helpful for the development of therapeutic agents for metabolic disorders. [78]. Previous studies have reported that SIRT1 upregulation protects against diabetes-induced liver toxicity [79]. In the present study, the downregulation of SIRT1 protein by EX-527 led to a protective effect against liver fibrosis in HFD-induced diabetic rats. Additionally, the protein expression of SIRT2, SIRT3 and SIRT4 was evaluated. SIRT2, SIRT3 and SIRT4 were significantly downregulated in HFD-induced diabetic rats compared with normal diet-fed rats; however, the expressions of SIRT2, SIRT3 and SIRT4 were increased after treatment with EX-527. Hence, effective therapeutic remedies are in high demand for the management of hepatic fibrosis and cirrhosis. The current study revealed that SIRT4 plays an important role in the protection against liver fibrogenesis, and the downregulation of SIRT1 reduces hepatic fibrogenesis in HFD-induced diabetic rats. These results suggest that the upregulation of SIRT4 is a promising approach to prevent fibrogenesis of liver. To the best our knowledge, this study is the first to show the protective effect of EX-527 via SIRT4 against hepatic fibrosis. Previous studies have shown that by blocking SIRT1 and SIRT2, renal interstitial fibroblast activation was inhibited [36]. Therefore, the role of SIRT4 in fibrogenesis could be the same in different tissues. However, the therapeutic role of other SIRT family members against hepatic fibrosis remains unclear. It would be interesting to explore whether there are specific functions of each subtype of SIRT in hepatic fibrosis.

Hepatocyte apoptosis or necrosis, as well as oxidative stress and lipid accumulation due to mitochondrial abnormality, ATP depletion, and membrane permeabilization are associated with hyperglycemia and hyperlipidemia in the liver [80,81,82]. Therefore, the preservation of a mitochondrial structure and functional integrity is the prime strategy in the treatment of liver fibrosis. Our study shows that oxidative stress and levels of inflammatory cytokines associated with liver fibrosis were restored with EX-527 treatment, which can ameliorate hepatic mitochondrial function impairment caused by HFD-induced liver fibrosis in diabetic rats. However, further preclinical and clinical investigations are required for addressing the mechanism of action of EX-527 against liver fibrosis.

## 5. Conclusions

In conclusion, this report demonstrates that EX-527 ameliorates the pathological progression of hepatic dysfunction in HFD-fed diabetic rats. The underlying mechanisms involve the inhibition of oxidative stress and inflammation, mitochondrial protection, the inhibition of TGF-β1 formation, and the reduction of apoptosis. Thus, our results provide a therapeutic clue for the clinical application of EX-527 as a potential hepatoprotective agent. Further research should be undertaken to elucidate the molecular mechanism of action of EX-527 in liver fibrosis.
